# Substantial Mitigation Potential for Greenhouse Gases Under High Water Levels in a Cultivated Peatland in the Arctic

**DOI:** 10.1111/gcb.70599

**Published:** 2025-11-10

**Authors:** Junbin Zhao, Cornelya F. C. Klütsch, Hanna Silvennoinen, Carla Stadler, David Kniha, Runar Kjær, Svein Wara, Mikhail Mastepanov

**Affiliations:** ^1^ Division of Environment and Natural Resources Norwegian Institute of Bioeconomy Research Ås Norway; ^2^ Department of Terrestrial Biodiversity Norwegian Institute for Nature Research Ås Norway; ^3^ Centro de Investigaciones en Física e Ingeniería del Centro de la Provincia de Buenos Aires Tandil Argentina; ^4^ Department of Ecoscience, Arctic Research Centre Aarhus University Roskilde Denmark; ^5^ Oulanka Research Station Oulu University Kuusamo Finland

**Keywords:** carbon balance, grassland, greenhouse gas, methane, nitrous oxide, photosynthesis, respiration, rewetting, water management

## Abstract

Drained cultivated peatlands are recognized as substantial global carbon emission sources, prompting the exploration of water level elevation as a mitigation strategy. However, the efficacy of raised water table level (WTL) in Arctic/subarctic regions, characterized by continuous summer daylight, low temperatures and short growing seasons, remains poorly understood. This study presents a two‐year field experiment conducted at a northernmost cultivated peatland site in Norway. We used sub‐daily CO_2_, CH_4_, and N_2_O fluxes measured by automatic chambers to assess the impact of WTL, fertilization, and biomass harvesting on greenhouse gas (GHG) budgets and carbon balance. Well‐drained plots acted as GHG sources as substantial as those in temperate regions. Maintaining a WTL between −0.5 and −0.25 m effectively reduces CO_2_ emissions, without significant CH_4_ and N_2_O emissions, and can even result in a net GHG sink. Elevated temperatures, however, were found to increase CO_2_ emissions, potentially attenuating the benefits of water level elevation. Notably, high WTL resulted in a greater suppression of maximum photosynthetic CO_2_ uptake compared to respiration, and, yet caused lower net CO_2_ emissions due to a low light compensation point that lengthens the net CO_2_ uptake periods. Furthermore, the long summer photoperiod in the Arctic also enhanced net CO_2_ uptake and, thus, the efficacy of CO_2_ mitigation. Fertilization primarily enhanced biomass production without substantially affecting CO_2_ or CH_4_ emissions. Conversely, biomass harvesting led to a significant carbon depletion, even at a high WTL, indicating a risk of land degradation. These results suggest that while elevated WTL can effectively mitigate GHG emissions from cultivated peatlands, careful management of WTL, fertilization, and harvesting is crucial to balance GHG reduction with sustained agricultural productivity and long‐term carbon storage. The observed compatibility of GHG reduction and sustained grass productivity highlights the potential for future paludiculture implementation in the Arctic.

## Graphical abstract

Elevating the water table in Arctic cultivated peatlands can substantially reduce greenhouse gas emissions, and even turn these systems into net GHG sinks. Fertilization enhanced biomass production but did not significantly affect CO_2_ or CH_4_ emissions, while biomass harvesting led to net carbon loss. The findings highlight the importance of integrated water, fertilization, and harvest management to balance climate mitigation and agricultural productivity in northern peatlands.

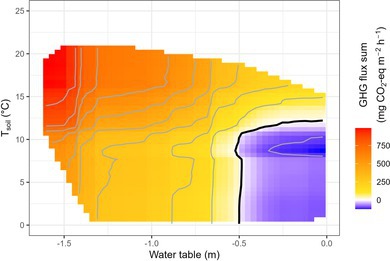

## 1 Introduction

Peatlands represent a significant portion of the global soil carbon (C) stock due to their anaerobic soil conditions, which slow down the carbon decomposition process. Since the 17th century, large areas of peatlands have been drained for purposes such as agriculture, forestry, mining, and urban development (Minasny et al. 2023). This practice exposes peat carbon to aerobic conditions, accelerating the decomposition process. As a result, drained peatlands have become “hotspots” for C emissions globally in the form of carbon dioxide (CO_2_), a potent greenhouse gas (GHG) (e.g., Kluge et al. 2008; Maljanen et al. 2010; Salm et al. 2009).

Elevating the water table level (WTL) in a drained peatland can restore anaerobic conditions, thereby reducing the decomposition rate and CO_2_ emissions (Darusman et al. 2023; Gunther et al. 2020; Liu et al. 2024). However, the effectiveness of elevated WTL varies across regions depending on many factors, such as soil conditions, vegetation types, and climate factors (Darusman et al. 2023). In addition, elevated WTL can also increase methane (CH_4_) emissions, which may partially or fully offset the reduction in CO_2_ emissions (Darusman et al. 2023). Therefore, the GHG balance must be carefully assessed to determine the optimal WTL for climate change mitigation under various environmental conditions.

Peatland cultivation typically involves fertilization, which facilitates crop growth and CO_2_ sequestration, but could also lead to significant emissions of nitrous oxide (N_2_O), another potent GHG (Kasak et al. 2022). Elevated WTL tends to reduce N_2_O emissions because the production of N_2_O is limited under anaerobic conditions (Lin et al. 2022; Liu et al. 2020). The overall GHG balance, considering CO_2_, CH_4_ and N_2_O emissions together, has been evaluated on cultivated peatlands in only a few studies (e.g., Beyer et al. 2015; Grønlund et al. 2006; Karki et al. 2015; Maljanen et al. 2001; Teh et al. 2011; Tiemeyer et al. 2016), largely due to the lack of simultaneous measurements of all GHGs. These studies represent only limited regions without coverage of higher latitudes, particularly those above 60° N. Importantly, their GHG measurements were typically conducted weekly or monthly while the diurnal variations of the fluxes and “hot moments” were hardly captured (Zhao et al. 2023). In fact, few studies have estimated GHG budgets on cultivated peatlands with sub‐daily data of all three main GHGs (Anthony and Silver 2021). To obtain an accurate budget estimation and a more comprehensive understanding of GHG fluxes, high temporal resolution flux data are needed.

Arctic/subarctic regions are characterized by subzero temperatures and snow cover for most of the year. Despite the extreme climate, several countries (e.g., Nordic countries) have a history of draining peatlands in these regions for agricultural use, such as cultivating grass as livestock feed (Unc et al. 2021). These activities may lead to significant CO_2_ emissions through peat decomposition; however, the GHG and C balance in these peatland systems has not been evaluated. It also remains uncertain whether a high WTL could reduce emissions in these regions. This is particularly important given the unique regional climate conditions, such as low temperatures and extended daylight periods. Investigating the GHG dynamics is crucial for understanding the role of these ecosystems in the global carbon cycle and developing effective mitigation strategies.

In this study, we conducted an experiment and measured sub‐daily GHG (CO_2_, CH_4_ and N_2_O) fluxes using an automatic chamber system at a cultivated peatland site in northern Norway during 2022–2023. To the best of our knowledge, this site represents the northernmost research location with active agricultural activities extending into the Arctic. Using plots with different water table levels and fertilization dosages, we aimed to answer the following questions: (1) Does a high WTL result in GHG neutrality in the cultivated peatland in the Arctic? (2) Is the CO_2_ emission (via ecosystem respiration) more sensitive to WTL changes than CO_2_ uptake (via photosynthesis)? (3) How do fertilization dosage and harvest frequency influence the GHG budget and C balance?

## 2 Methods and Materials

### 2.1 Study Site and Experiment Design

The site is a cultivated peatland in the Pasvik Valley (69°28′33″ N, 29°59′24″ E). Despite its Arctic location, the site exhibits a subarctic climate (Iturbide et al. 2020) due to the influence of Atlantic air masses. In this study, we use “Arctic” to denote the geographical location and “subarctic” to characterize the climate. It has a growing season of 4–5 months. The annual mean temperature and precipitation are 0.5°C and 480 mm, respectively. The peatland was drained in the 1970s and cultivated with meadow fescue ( *Festuca pratensis* ) mixed with timothy ( *Phleum pratense* ). Water table levels vary significantly across different locations depending on the distance to the drainage and water flow. Along the water level gradient, we established 5 plots (~50 m^2^ each) to represent a range of water conditions from well‐drained to wet. Each plot was divided into two subplots, where low (350 kg ha^−1^, representing local practice) and high (700 kg ha^−1^) fertilization dosages were applied, respectively (see Figure S1 for the design overview). Fertilization dosages were administered in two separate applications each year (2022: May 12 and August 9; 2023: May 25 and July 12). The fertilizer used is Fullgjødsel “18‐3‐15” (Yara International ASA, Oslo, Norway). The grass was harvested manually (cut at stubble height) once in 2022 on August 9th and twice in 2023 on July 11 and September 3. The fresh and dry weights of the harvested biomass were measured following each harvest. The C content was calculated as 43% of the dry biomass based on the synthesis for herbaceous plants reported by Ma et al. (2018). Soils at the site, on average, contain 46% carbon (organic), 2.8% nitrogen with a bulk density of 0.23 g cm^−3^. Peat depths vary from 1.05 to 1.80 m. The soil pH is ~6.2. The von Post humification is estimated to be between H3 and H6.

### 2.2 GHG Flux Measurements

Fluxes of CO_2_, CH_4_ and N_2_O were measured using an automatic chamber system (see Figure S2 and Mastepanov (2025) for the chamber design). The system consists of multiple custom‐made transparent chambers (60 × 60 × 60 cm; polycarbonate glass) that are programmed to carry out flux measurements in sequence. For each measurement, the lid of a chamber was closed (controlled pneumatically) and air from the chamber headspace was routed to a Picarro G2508 analyzer (Picarro Inc., Santa Clara, CA, USA) for gas analysis (1 Hz). Each measurement lasted for 16 min, including a 3‐min flushing period both before and after the measurement. Three chambers were installed in each subplot, making a total of 30 chambers. Thus, each chamber was measured at 8 h intervals throughout the snow‐free seasons (mainly May–October). During 2022–2023, we collected data from 21,410 measurements, each encompassing fluxes of CO_2_, CH_4_, and N_2_O. The transparent chambers increased air temperature inside by 0.4°C ± 1.7°C (SD) compared to the ambient temperature during the measurement period. The warming effect is most significant around noon (11:00–13:00), reaching 1.3°C ± 1.6°C. The measurements were powered by a system that consists of solar panels, a windmill and a diesel generator. Due to power and technical failures in 2022, a few flux data gaps were present with the largest gap being between July 20 and August 10 (Figures S3–S5).

### 2.3 Environmental Data

Soil temperatures and moisture were monitored at 2, 5, 10, 20, 50, and 75 cm depths in each plot using the Stevens Water Hydraprobe (Stevens Water Monitoring Systems Inc., Portland, OR, USA). Water table levels in each plot were observed using the Seametrics PT12 (Seametrics Inc., Kent, WA, USA). We also used hourly data of air temperature, relative humidity, precipitation, wind speed and global radiation from a weather station (69°27′18″ N, 30°02′28″ E) which is ~3 km away from the study site (frost.met.no).

### 2.4 Data Processing and Analysis

GHG fluxes were calculated based on linear regressions of the changes in gas concentrations over the chamber closure period. Studies using automatic chambers have reported substantial overestimations of fluxes under stable atmospheric conditions (typically at night) on porous peat soils when measurement durations are shorter than 2 min (Koskinen et al. 2014; Lai et al. 2012). These studies recommend extending measurement durations to capture more linear and stabilized gas concentration changes toward the end of the measurement period and thereby reduce bias. To address this issue, we applied a dynamic window approach in our flux calculations, selecting the best linear fit from the latter part of each measurement. On average, ~8 min of data was included in the final flux estimates. For quality control, data with *R* ^2^ > 0.8 were assigned a quality flag of “0”. Low fluxes, often near the analyzer's detection limit, can produce noisy concentration patterns (low R^2^) at high sampling frequencies. Despite their minimal contribution to the seasonal flux budgets, these small fluxes are important for statistical analyses. Therefore, we assigned a quality flag of “1” to fluxes that were lower than ±100 mg CO_2_ m^−2^ h^−1^, ±0.1 mg CH_4_ m^−2^ h^−1^ or 0.2 mg N_2_O m^−2^ h^−1^. The remaining data were marked as “2”. Only data marked as “0” and “1” were included for further analysis. Consequently, 5% of CO_2_ data, 0.2% of CH_4_ data, and 7% of N_2_O data were excluded. Unless otherwise indicated, positive flux values denote emissions, while negative values denote uptakes.

We used mixed‐effects linear models to investigate the effects of plots, fertilization treatments, and study years, on CO_2_, CH_4_, N_2_O fluxes and dry biomass. The interactions between plots, fertilization levels, and study years were included as main effects. If the interaction terms were not statistically significant (*p* > 0.05), they were replaced by the individual effects in the model. Chamber ID and measurement date were included as random effects to account for spatial variances and the repeated measures nature of the data. The “lme4” (v1.1–36) (Bates et al. 2015) and “lmerTest” (v3.1–3) (Kuznetsova et al. 2016) packages in R were used for the model fitting.

Random forest (RF) models were developed to examine the relationships between GHG fluxes and environmental factors (primarily temperature and water table level), and to perform data gap‐filling for estimating seasonal flux budgets. Specifically, we developed three separate RF models for CO_2_, CH_4_, and N_2_O fluxes, with each model utilizing 13 predictors: plot ID (as a categorical variable: 1–5), fertilization treatment (as a categorical variable: high vs. low), global radiation, water table level (WTL), wind speed, vapor pressure deficit (VPD), day of year, precipitation, soil temperature at 5 and 50 cm depths, air temperature, days since harvest, and days since fertilization. To address potential bias in predicting extreme values, data points exceeding the 95th percentile were oversampled. For CH_4_ fluxes, measurements exceeding 10 mg CH_4_ m^−2^ h^−1^ (*n* = 7 or 0.03%) were excluded from model training but included later in the budget calculations.

RF model tuning involved an initial run to determine an appropriate number of trees (“ntree”). As model output stabilized at “ntree” values above ~450, “ntree” was set to 600 for further tuning. Grid search within the ranges 100–1800 (with 100 intervals) and 1–12 was employed to optimize “maxnodes” (maximum number of terminal nodes) and “mtry” (number of variables considered at each split), respectively. Ten‐fold cross‐validation was performed, and data independence was ensured by grouping observations within each chamber ID into separate folds. Models with the lowest root mean square error (RMSE) were selected. The R packages “randomForest” (v4.7–1.2) (Liaw and Wiener 2002) and “caret” (7.0–1) (Kuhn 2008) were used for the RF model training.

The final RF models were used to estimate hourly fluxes, filling data gaps for seasonal budget calculations (May–October). Bootstrap sampling was employed to assess uncertainty, with predictions generated from individual trees (ntree = 600). Uncertainties associated with bootstrap sampling and field replicates were calculated separately and summarized in Table S1. Global warming potential over 100 years (GWP_100_) was applied to convert CH_4_ and N_2_O fluxes into CO_2_‐equivalents (Neubauer 2021), using factors of 27 and 273 for CH_4_ and N_2_O, respectively (IPCC 2021). Partial dependence plots, generated using the “pdp” package (v0.8.2) (Greenwell 2017) in R, were created to investigate the effects of WTL and soil temperature on fluxes. To compare with the general relationships between fluxes and WTL reported by Tiemeyer et al. (2020) and Evans et al. (2021), we also calculated annual budgets for CO_2_ and CH_4_ fluxes. For periods outside of the measurement seasons (from November to the following April), we assumed CO_2_ flux to be 1.5 g C m^−2^ month^−1^ following Evans et al. (2021) and CH_4_ flux to be 15% of the annual total as per Abdalla et al. (2016).

To examine the effect of WTL on CO_2_ flux components, data were grouped into 0.3 m intervals from −1.5 to 0 m. Modeled fluxes were excluded in this analysis. The Michaelis–Menten rectangular hyperbola (Falge et al. 2001) was used to fit data for each group:

(1) $${\mathrm{CO}}_{2}\;\text{flux}=\frac{\alpha \times R_{g}\times {\mathrm{GPP}}_{\max}}{\alpha \times R_{g}+{\mathrm{GPP}}_{\max}}+R_{d}$$

where α is light use efficiency (mg CO_2_ m^−2^ h^−1^ per W^−1^ m^−2^), *R*_*g*_ is global radiation (W m^−2^), GPP_max_ is maximum gross primary production (mg CO_2_ m^−2^ h^−1^), and *R*_*d*_ is dark respiration (mg CO_2_ m^−2^ h^−1^). The “minpack.lm” package (v1.2–4) was used for curve fitting (Elzhov et al. 2016). Light compensation point (LCP), the light intensity at which CO_2_ flux equals zero, was calculated from the fitted parameters:

(2) $$\mathrm{LCP}=\frac{R_{d}\times {\mathrm{GPP}}_{\max}}{\alpha \times \left({\mathrm{GPP}}_{\max}-R_{d}\right)}$$

Comparisons of GPP_max_, *R*_*d*_, and LCP across WTL groups were conducted to assess their individual responses to WTL changes. Parameter uncertainties were estimated through 300 bootstrap iterations. All data processing and analysis were performed using the R program 4.4.2 (R Development Core Team 2024). The data and code are publicly available on Zenodo (DOI: https://doi.org/10.5281/zenodo.17423612).

## 3 Results

### 3.1 Environmental Factors

**FIGURE 1:**
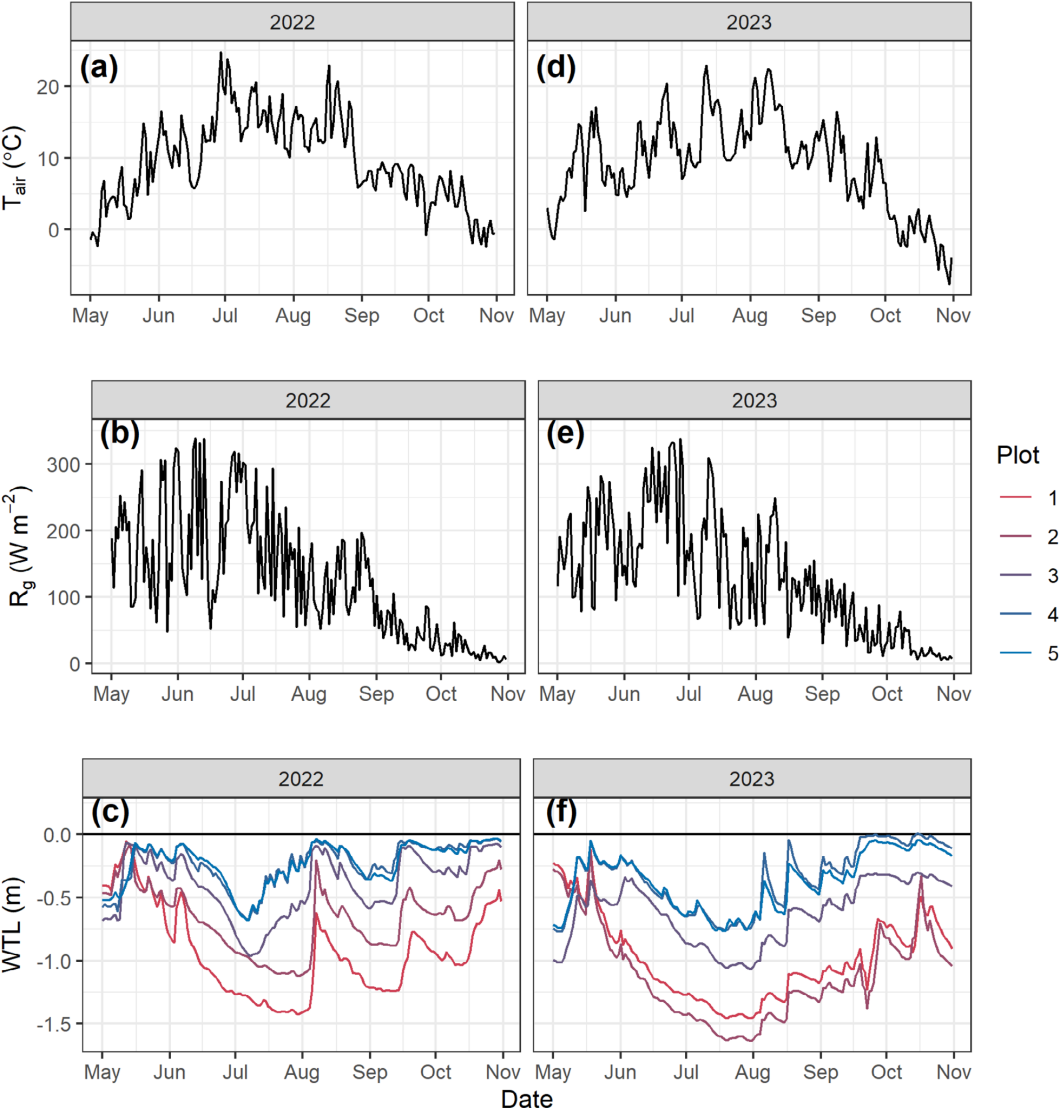
Daily mean air temperature (Tair, a, d), global radiation (R_g_, b, e) and water table level (WTL, c, f) variations during May–October in 2022 (a–c) and 2023 (d–f).

Over the measurement period (May–October), air temperature exhibited a seasonal pattern with values reaching ~15°C during June–August with peak values reaching approximately 23°C in both years (Figure 1a,d). The temperature dropped below 0°C from mid‐ or early October. Global radiation also displayed a pronounced seasonal pattern, peaking around 300 W/m^2^ in June (Figure 1b,e). Water table levels fluctuated throughout the growing season, with variations observed across the different plots. Plots 1 and 2 exhibited the deepest WTL with the lowest values (late June) reaching −1.4 m in 2022 and −1.6 m in 2023 (Figure 1c,f). The WTL was the highest in plots 4 and 5 with the lowest values being approximately −0.7 m in July for both years.

### 3.2 GHG Fluxes

Throughout the study years, the effects of different plots and fertilization dosage were different for fluxes of the three GHGs (Table 1). For the CO_2_ flux, the effect of fertilization was not significant (*p* = 0.61), while the flux was significantly affected by the plot (*p* < 0.01) and study year (*p* < 0.01). No significant interactions were observed among the factors (*p* > 0.05). For the CH_4_ flux, the interaction between fertilization and plot was significant (*p* < 0.01), suggesting that the effect of fertilization on methane emissions varied across the different WTLs. The study year also showed a significant effect on the CH_4_ flux (*p* < 0.01). The N_2_O flux was significantly affected by fertilization (*p* < 0.01) and study year (*p* < 0.01) but was not different among the plots (*p* = 0.32). No significant interactions were observed among the factors (*p* > 0.05).

**TABLE 1:** Analysis of variance (ANOVA) of linear mixed effect models for CO_2_ flux (mg CO_2_ m^−2^ h^−1^), CH_4_ flux (mg CH_4_ m^−2^ h^−1^), N_2_O flux (mg N_2_O m^−2^ h^−1^) and dry biomass (t ha^−1^).

Model	Effect	SS	NumDF	DenDF	*F*	*p*
CO_2_ flux	Fertilization	5.81 × 10^4^	1	24	0.27	0.607
	Plot	9.07 × 10^6^	4	25	10.59	< 0.001
	Year	10.43 × 10^7^	1	6063	48.72	< 0.001
CH_4_ flux	Fertilization	3.65	1	36	3.81	0.059
	Plot	62.53	4	46	16.34	< 0.001
	Year	7.96	1	2775	8.32	0.004
	Fertilization: Plot	19.41	4	31	5.07	0.003
N_2_O flux	Fertilization	0.22	1	25	8.87	0.006
	Plot	0.12	4	26	1.24	0.318
	Year	1.27	1	5713	52.01	< 0.001
Dry biomass	Fertilization	17.34	1	24	12.17	0.002
	Plot	15.27	4	24	2.68	0.056
	Year	0.10	1	29	0.07	0.791

*Note:* Chamber ID and the measurement date are included as random effects. Note that the interaction term (Fertilization: Plot) was found to be significant only in the CH_4_ flux model (*p* < 0.05) and, thus, models for CO_2_ flux, N_2_O flux and biomass were made without interactions.

Abbreviations: DenDF, denominator degrees of freedom; NumDF, numerator degrees of freedom; SS, type III sums of squares with Satterthwaite approximation for degrees of freedom.

### 3.3 Random Forest Models

**FIGURE 2:**
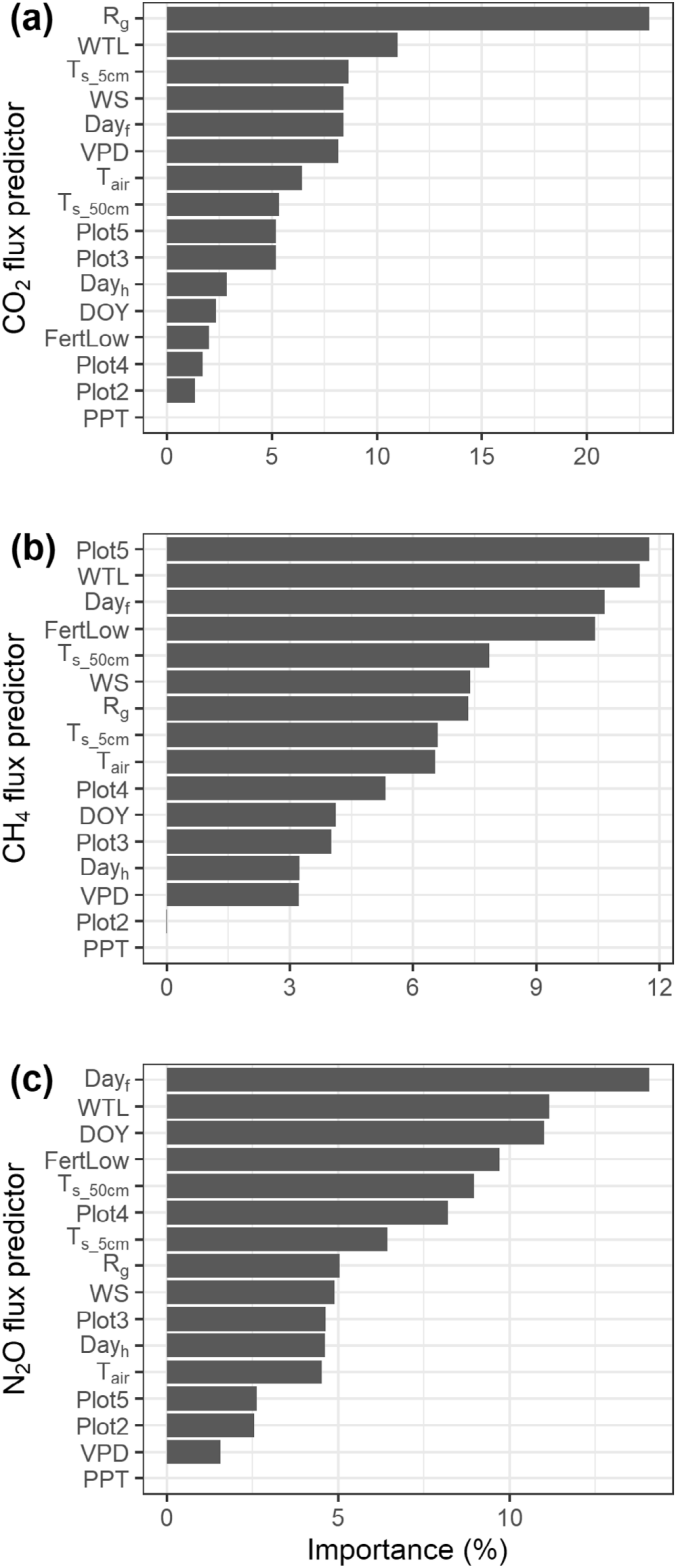
The importance of each variable as a predictor in the model for CO_2_ (a), CH_4_ (b), and N_2_O (c) fluxes. R_g_, global radiation. WTL, water table level. T_air_, air temperature. T_s_5cm_, soil temperature at 5 cm depth. T_s_50cm_, soil temperature at 50 cm depth. WS, wind speed. VPD, vapor pressure deficit. DOY, day of year. PPT, precipitation. Day_f_, days since the last fertilization. Day_h_, days since the last harvest. FertLow, the low fertilization treatment.

The final RF models showed RMSEs of 680, 0.33 and 0.18 mg gas m^−2^ h^−1^, respectively, for CO_2_, CH_4_ and NO_2_ fluxes (Table 2). The models explained 78%–83% of the flux variances. As to predictors, global radiation contributed to 23% of the explanatory power of the CO_2_ flux model, followed by WTL (11%), soil temperature at 5 cm (8%), wind speed (8%), days since fertilization (8%) and VPD (8%) (Figure 2a). For the CH_4_ flux model, plot 5 (the wettest plot) and WTL together contributed 23% of the explanatory power, followed by days since fertilization (11%) and the fertilization treatment (10%) (Figure 2b). N_2_O fluxes were mostly explained by days since fertilization (14%), followed by WTL (11%) and day of year (11%), and the fertilization treatment (10%) (Figure 2c).

**TABLE 2:** Statistics of the final random forest models for CO_2_/CH_4_/N_2_O fluxes (mg gas m^−2^ h^−1^).

Model	maxnodes	mtry	RMSE	*R* ^2^	*N*
CO_2_ flux	1500	3	680	0.82	22,759
CH_4_ flux	1000	3	0.33	0.83	23,319
N_2_O flux	1500	3	0.18	0.78	20,063

*Note:* See Figure S6 for model predictions versus observed fluxes.

Abbreviations: maxnodes, maximum number of terminal nodes for the trees; mtry, number of variables to consider when making each split in the trees; *N*, number of samples used for model training; RMSE, root mean square error from the 10‐fold cross validation.

**FIGURE 3:**
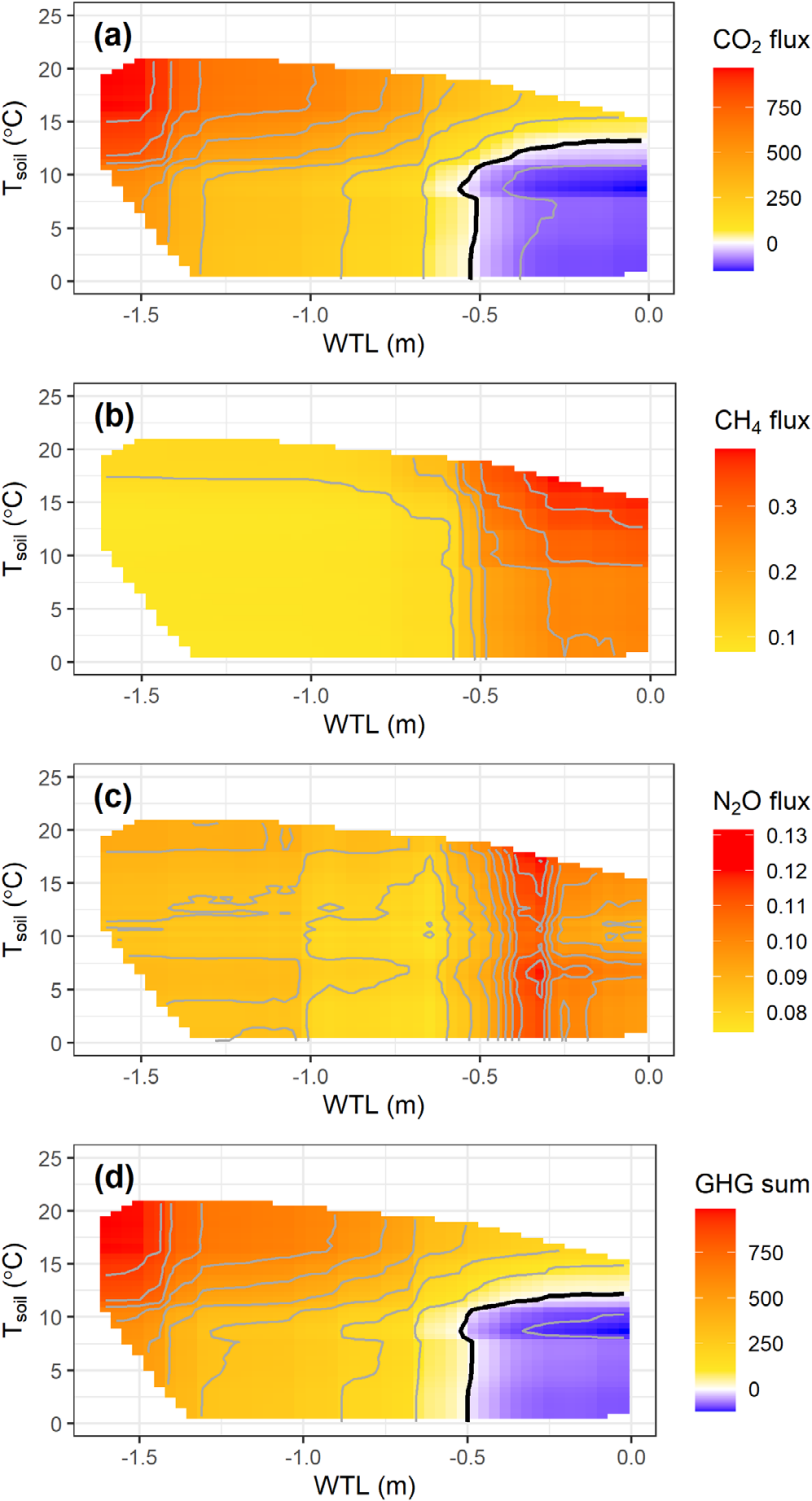
Partial dependence plots showing the fluxes of CO_2_ (a; mg CO_2_ m^−2^ h^−1^), CH_4_ (b; mg CH_4_ m^−2^ h^−1^), N_2_O (c; mg N_2_O m^−2^ h^−1^), and sum of all three gases (d; mg CO_2_‐eq m^−2^ h^−1^) in relation to water table level (WTL) and soil temperature at 5 cm depth (T_soil_). The black contour lines indicate the zero points for the CO_2_ flux and sum of CO_2_‐equivalent GHG fluxes.

The partial dependence plot reveals that CO_2_ emissions were the highest under dry and warm conditions (Figure 3a). The increase of WTL above −0.5 m could turn the ecosystem into a net sink for CO_2_ when soil temperature < 10°C. Further temperature increases could significantly raise the WTL threshold for CO_2_ compensation, potentially preventing the ecosystem from becoming a carbon sink when soil temperatures exceed 15°C. It is noted that the CO_2_ flux is sensitive to light conditions, and these results reflect the CO_2_ flux under the average global radiation (132 W m^−2^) over the observation periods. The CH_4_ flux was generally very low (≤ 0.1 mg CH_4_ m^−2^ h^−1^) when the WTL was below −0.6 m (Figure 3b). The partial dependence plot, representing the average CH_4_ flux, did not capture the occasional small CH_4_ uptake observed in the raw data at the low WTL. The CH_4_ flux became higher than 0.3 mg CH_4_ m^−2^ h^−1^ when soil temperature was greater than 15°C and WTL greater than −0.5 m. For the N_2_O flux, WTL showed a nonlinear impact where flux values were the lowest between −1 and −0.5 m (< 0.08 mg N_2_O m^−2^ h^−1^) and the highest between −0.4 and −0.3 m (Figure 3c). Soil temperature generally exhibited a weak positive impact on N_2_O flux. Despite the inclusion of CH_4_ and N_2_O fluxes, the sum of CO_2_‐equivalent GHG fluxes was primarily influenced by CO_2_ flux, exhibiting a similar pattern of variation in response to temperature and WTL as observed for CO_2_ emissions (Figure 3d).

### 3.4 Components of the CO_2_ Flux

**FIGURE 4:**
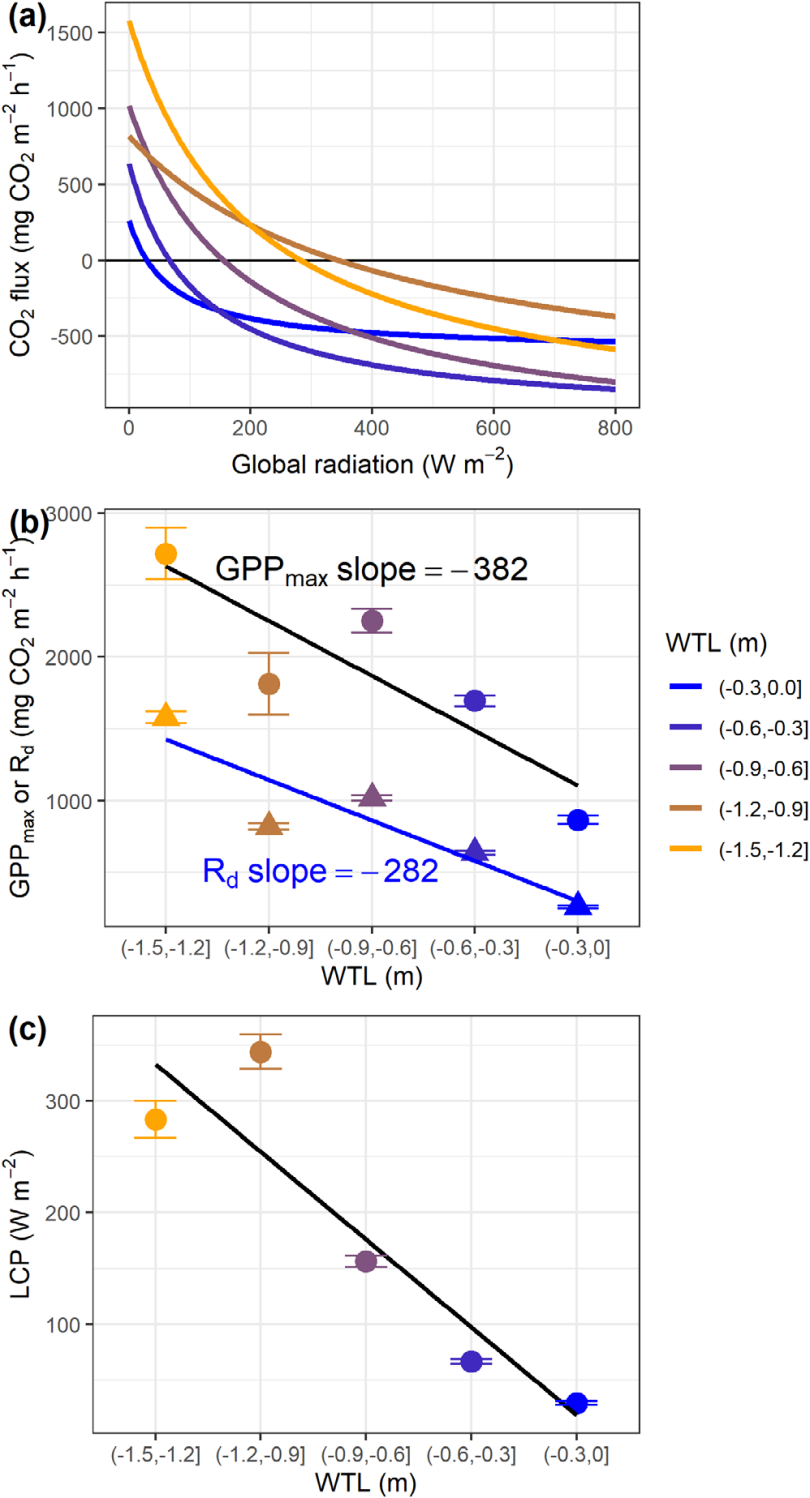
The responses of CO_2_ fluxes to global radiation (a) and the corresponding parameters GPP_max_ (maximum gross primary production, circle), R_d_ (dark respiration, triangle) (b) and light compensation points (LCP) (c) at different water table levels (WTL). The points are mean ± SD of 300 bootstrap samples. Linear regression lines are included in panels (b) and (c) to aid visualization. Note that positive values are used for GPP_max_ to facilitate comparison with R_d_.

The CO_2_ flux showed different responses to light change over the WTL gradients (Figure 4a). From the extracted parameters, we found that both dark respiration (*R*_*d*_) and GPP_max_ decreased as WTL increased, with a larger slope in GPP_max_ than in *R*_*d*_ (Figure 4b). The light compensation points also dropped from ~300 W m^−2^ under WTL of less than equal to −0.9 m to ~30 W m^−2^ under WTL of greater than −0.3 m (Figure 4c).

### 3.5 Seasonal GHG Budget

**FIGURE 5:**
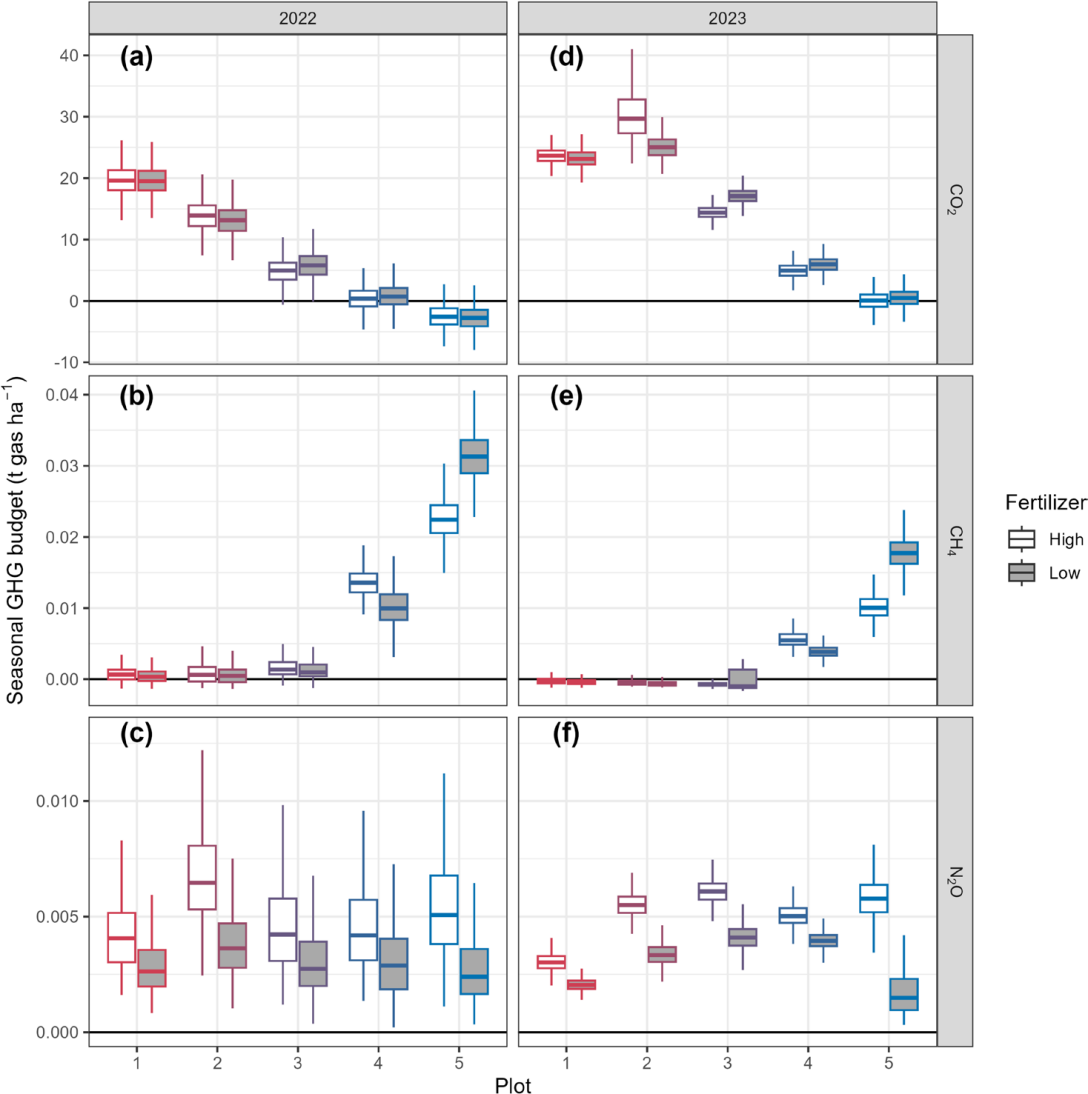
Seasonal budgets (May–October) of CO_2_ (a, d), CH_4_ (b, e) and N_2_O (c, f) fluxes from the 5 plots in 2022 (a–c) and 2023 (d–f). Different fertilizer dosages are shown using white (high) and grey boxes (low). The box represents the interquartile range (IQR) between the first and third quartiles, with a line indicating the median, and whiskers extending to values within 1.5 times the IQR. *N* = 1800 (3 replicates × 600 bootstrap samples).

The seasonal budget of CO_2_ flux showed a general decrease from a substantial source in plot 1 to a small sink/neutral in plot 5 with no obvious differences between high and low fertilization treatments (Figure 5a,d). The CO_2_ emissions were generally 3–10 t CO_2_ ha^−1^ higher in 2023 than in 2022. For the CH_4_ flux budget, plots 1–3 were generally neutral while emissions were noticeably present in plots 4 and 5 (Figure 5b,e). The highest CH_4_ emissions were present in plot 5 under the low fertilization treatment. The CH_4_ emissions were generally higher in 2022 than in 2023, corresponding to higher WTLs in 2022 (Figure 1c,f). The N_2_O flux budget did not show a clear pattern among the 5 plots but the budgets were generally higher in the high fertilization treatment (Figure 5c,f). In 2023, plots 3 and 4 exhibited higher N_2_O emissions than in 2022 while emissions from other plots were similar between the 2 years.

### 3.6 Biomass From Harvests

**FIGURE 6:**
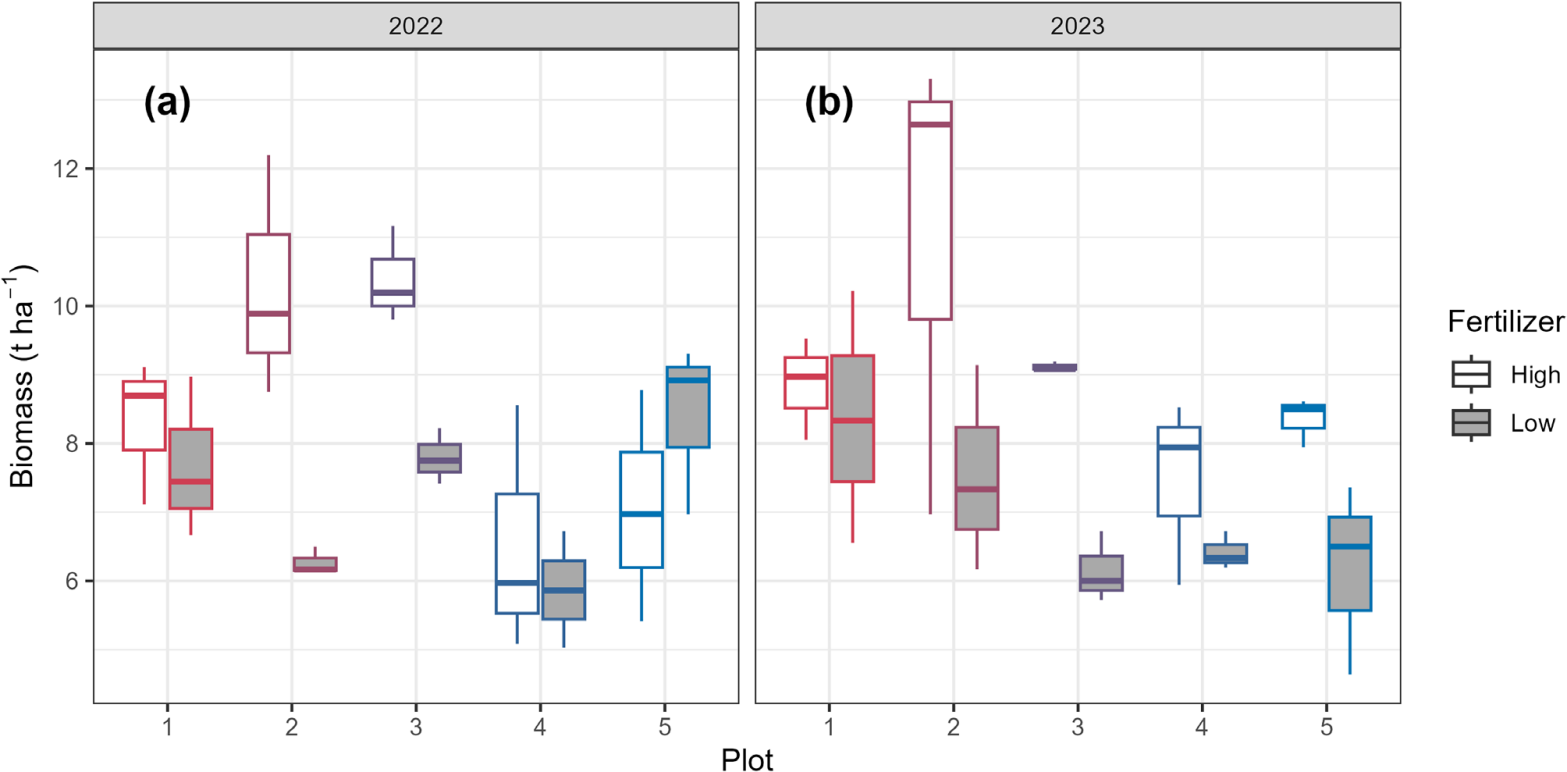
Harvested dry biomass from the 5 plots in 2022 (a) and 2023 (b). Different fertilizer dosages are shown using white (high) and grey boxes (low). The box represents the interquartile range (IQR) between the first and third quartiles, with a line indicating the median, and whiskers extending to values within 1.5 times the IQR (*N* = 3).

The harvested biomass showed a slight decrease from plot 1 to 5 (Figure 6) but the differences were not significant according to the linear mixed‐effect model (*p* = 0.06) (Table 1). There was more biomass harvested from the high fertilization subplots than from the low fertilization ones (*p* < 0.05), as shown in plots 2 and 3 in 2022 and in plots 2–5 in 2023. No significant interaction was present between the plot and fertilization treatment (*p* = 0.08). Despite the different harvest frequencies between 2022 (once) and 2023 (twice), the total harvested biomass was not different between the 2 years (*p* = 0.79).

### 3.7 Balance of GHG and C

**FIGURE 7:**
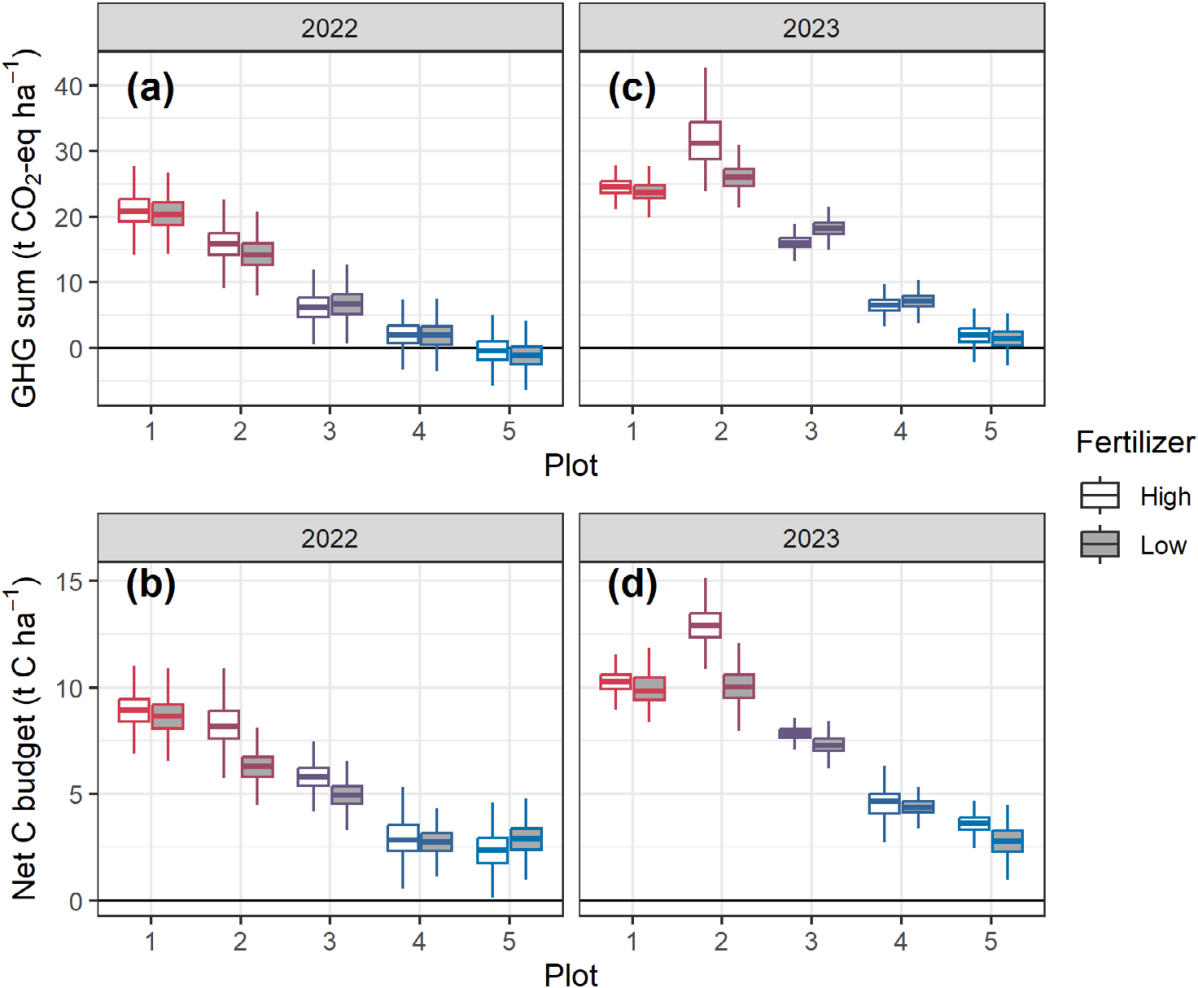
Sum of CO_2_‐equivalent GHG fluxes (a, c) and net C budget (b, d) from the 5 plots in May–October of 2022 (a, b) and 2023 (c, d). Different fertilizer dosages are shown using white (high) and grey boxes (low). The box represents the interquartile range (IQR) between the first and third quartiles, with a line indicating the median, and whiskers extending to values within 1.5 times the IQR. *N* = 1800 (3 replicates × 600 bootstrap samples).

Similar to CO_2_ flux budget, the sum of CO_2_‐equivalent GHG fluxes decreased from plot 1 to 5, with plot 1 being a substantial source and plot 5 being GHG neutral (Figure 7a,c). Accounting for the biomass removal from the ecosystem, all plots became sources of C, with the largest release observed in plots 1 and 2, reaching up to ~13 t C ha^−1^ in 2023 (Figure 7b,d). Even the wettest plots (4 and 5) lost at least ~2.5 t C ha^−1^ for each season.

## 4 Discussion

**FIGURE 8:**
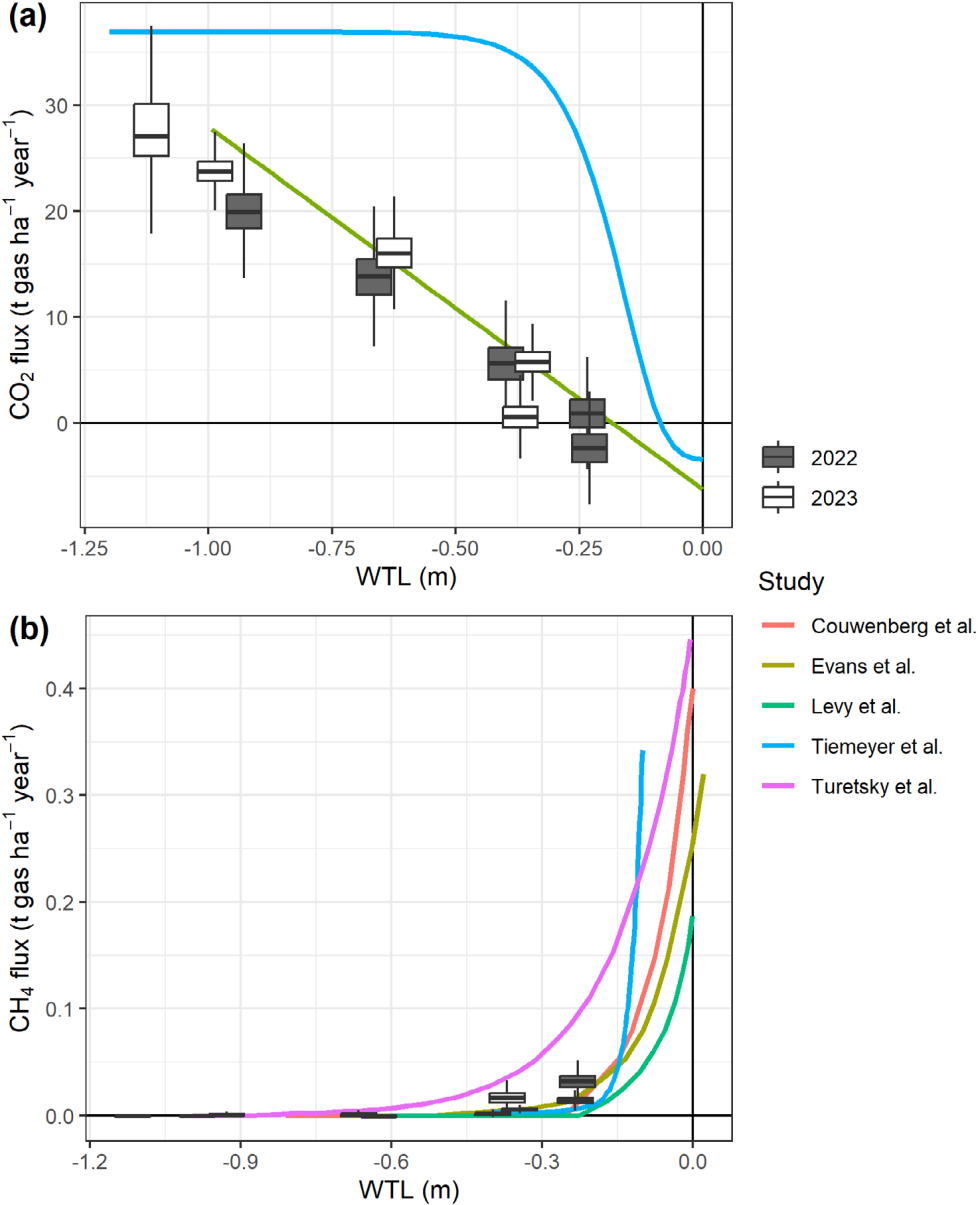
Estimations of CO_2_ (a) and CH_4_ (b) annual budgets from the 5 plots in 2022 and 2023 as a function of water table level (WTL) in comparison with relationships (solid lines) from other studies (Couwenberg et al. 2011; Evans et al. 2021; Levy et al. 2012; Tiemeyer et al. 2020; Turetsky et al. 2014). The box represents the interquartile range (IQR) between the first and third quartiles, with a line indicating the median, and whiskers extending to values within 1.5 times the IQR. *N* = 3600 (3 replicates × 600 bootstrap samples × 2 fertilization dosages).

Field observations of simultaneous CO_2_, CH_4_ and N_2_O fluxes are scarce in cultivated peatlands, and high‐latitude regions, especially those within the Arctic Circle, are particularly underrepresented (Zhao et al. 2023). Our study, conducted at the northernmost agricultural site located in the Arctic, to our knowledge, addresses this critical knowledge gap. While the climate is classified as subarctic, the site exhibits distinct radiation patterns (continuous daylight in summer), short growing seasons and low temperatures, which are typical in geological Arctic regions. Our findings reveal that drained peatlands in the Arctic can emit substantial CO_2_, comparable to those at lower latitudes. Maintaining a WTL between −0.5 and −0.25 m effectively reduces CO_2_ emissions while limiting CH_4_ and N_2_O contributions. Notably, the observed CO_2_ flux‐WTL relationship closely mirrored findings by Evans et al. (2021) for boreal and temperate cultivated peatlands (Figure 8a), highlighting the robustness of this relationship across diverse climate zones. However, the nonlinear relationship reported by Tiemeyer et al. (2020) did not fit our data well, likely because their analysis included sites with other land uses (e.g., forest, shrubland). Although the CH_4_ budgets were marginal even at our wettest plots, a higher mean WTL of > −0.2 m may exponentially increase CH_4_ emissions (Couwenberg et al. 2011; Evans et al. 2021; Tiemeyer et al. 2020; Turetsky et al. 2014), potentially diminishing the climate benefits of water level elevation. Importantly, we observed that wet conditions not only reduced GHG emissions but also facilitated the transformation of the ecosystem into a GHG sink, all while sustaining good grass productivity. These results demonstrate the promising potential for future paludiculture implementation even in the Arctic, underscoring the necessity for further research to optimize management strategies to the unique ecological conditions of these regions.

Through light response curves, this study reveals, for the first time, that high WTL suppressed maximum CO_2_ uptake via photosynthesis even more than the CO_2_ emission through respiration. High WTL can create anoxic soil conditions that decrease hydraulic conductivity and impose stress on plants. In response, plants typically close stomata and/or reduce carboxylation and electron transport (Pezeshki et al. 1996; Zhao et al. 2021; Zhao et al. 2018), consequently lowering photosynthetic rate. This result seemingly contradicts the established association of elevated WTL with reduced net CO_2_ emissions; however, the observed emission reduction is primarily attributed to a lower light compensation point under wet conditions, thereby decreasing the light threshold required for the ecosystem to function as a C sink. This reduced light compensation point is the key mechanism that determines the mitigation effect of elevated water levels and thus, is crucial to be included in process models to achieve more accurate emission estimations. Furthermore, the long Arctic summer daylight increased net CO_2_ uptake by up to 1.9 t CO_2_ ha^−1^ season^−1^ (compared to assuming no global radiation between 20:00 and 4:00), and thus substantially enhanced the efficacy of CO_2_ mitigation. Given the importance of vegetation, future analyses incorporating vegetation data (e.g., NDVI) may yield further insights into explaining and predicting GHG fluxes.

Temperature plays a significant role in driving CO_2_ and CH_4_ fluxes. Specifically, when interacting with WTL, the effectiveness of WTL in turning the ecosystem into a CO_2_ sink diminishes as soil temperature exceeds 12°C. Higher temperatures enhance microbial metabolism, thereby increasing C decomposition rates and methanogenic activity (Bridgham and Richardson 1992), leading to greater CO_2_ and CH_4_ releases. This suggests that raising water levels may be more effective in colder regions than in warmer areas. Future warming climate could also dampen the mitigation efficacy, which needs to be further investigated. Our results highlight the potential limitations of WTL management as a sole mitigation strategy in warmer regions, where temperature‐driven C losses can counteract the benefits of higher WTL. We suggest considering temperature as a key factor in peatland management and restoration efforts aimed at mitigating GHG emissions. In addition, managing the water table is challenging in practice, particularly in highly degraded peat soils where subsidence and altered hydrological properties complicate water retention. Although raising the water table is widely recognized as an effective measure to reduce CO_2_ emissions, its implementation is often constrained by technical, hydrological, and land‐use limitations. Therefore, more studies focusing on water management techniques and strategies are needed to achieve more effective emission mitigations (Boonman et al. 2022; Triadi 2020).

A limitation of our study is that only transparent chambers were used, and, thus, dark respiration was not explicitly measured. While we did capture a considerable number of fluxes under dark conditions (*N* = 5729; global radiation < 10 W m^−2^), these were relatively few during the peak summer months of May–July (*N* = 1407; Figure S7). This imbalance led to a dataset skewed toward light conditions, which in turn caused our RF model to underestimate CO_2_ emissions by approximately 15% under dark conditions. This underestimation was particularly significant under low WTL, where emissions tend to be higher. Nevertheless, since dark hours represent only ~30% of the growing season, the resulting bias in our seasonal CO_2_ budget is likely minor (< 5%). N_2_O flux exhibited a nonlinear relationship with WTL, demonstrating a greater sensitivity to WTL than to temperature, with peak flux values observed within the WTL range of −0.4 to −0.3 m. Similar patterns were also found in Parn et al. (2018), who reported an optimal soil water content of ~50% for N_2_O emissions. At our site, N_2_O fluxes are largely driven by fertilization activities, indicating the importance of substrate availability. A temperate study on cultivated peatlands suggested that hot moments (fluxes that are > 4 × SD of mean) account for 57% of the annual N_2_O emissions (Anthony et al. 2023). In our measurements, hot moments accounted for up to 34% of the seasonal budget, underscoring the importance of the hot moments at high latitudes. Therefore, we suggest that budget estimations based on low temporal resolution flux measurements may fail to capture N_2_O hot moments and thus, significantly underestimate N_2_O emissions. While our RF model had a satisfactory performance for modeling N_2_O fluxes, it still underestimates many fluxes that are > 1.5 mg N_2_O m^−2^ h^−1^ (Figure S6). Future research that investigates the drivers and improves the N_2_O flux prediction is still crucial.

While fertilization dosage did not influence the emissions of CO_2_ and CH_4_, a higher dosage enhanced the biomass production. On the other hand, we found that biomass harvesting can lead to net C loss even at high WTL, contributing to soil C depletion and land degradation. It is important to note that our C balance estimates are conservative, as they do not account for lateral export, such as dissolved organic C (Strack et al. 2008), which could further enhance the C deficit. The harvest frequency (1 cut in 2022 vs. 2 cuts in 2023) appeared to influence GHG fluxes, likely through changing the number of photosynthetic leaves; however, the total biomass yield was not affected by the harvest frequency. These results highlight the need for careful consideration of both water management and biomass harvesting strategies that not only reduce GHG emissions but also ensure the long‐term sustainability of the ecosystem's C storage capacity. A holistic approach, such as an adaptive harvest scheme, needs to be developed to consider the trade‐offs between C sequestration, biomass use, and overall ecosystem health.

At our site, the contrasting WTL across the 5 plots represents typical spatial variations of hydrological conditions in a single drained field. The heterogeneity of the GHG fluxes across our field highlights the importance of considering small scale and site‐specific conditions for estimating GHG emissions and improving agricultural management. For national GHG accounting, the IPCC suggested a Tier 1 CO_2_ emission factor of 29 t CO_2_ ha^−1^ year^−1^ for drained croplands on organic soils in boreal regions (Hiraishi et al. 2014). However, our study revealed substantial spatial heterogeneity in CO_2_ emissions within a single drained field, ranging from −2 to 27 t CO_2_ ha^−1^ year^−1^ across different locations. Moreover, with ~50 years of cultivation history, the peat soil at our site is less degraded (von Post humification: H3‐6) compared to many sites that have been drained and cultivated for over a century, where lower emissions are expected due to the depletion of labile C pools. Such potential disparities in emissions among sites, or even within a single site, underscore the limited representativeness of the IPCC Tier 1 emission factor and necessitate the development of more accurate Tier 2 or 3 methodologies for countries with extensive peatland distribution.

## Author Contributions

**Junbin Zhao:** conceptualization, data curation, formal analysis, funding acquisition, investigation, methodology, project administration, resources, software, supervision, validation, visualization, writing – original draft, writing – review and editing. **Cornelya F. C. Klütsch:** investigation, methodology, project administration, supervision, writing – review and editing. **Hanna Silvennoinen:** conceptualization, funding acquisition, methodology, project administration, writing – review and editing. **Carla Stadler:** data curation, investigation, methodology, writing – review and editing. **David Kniha:** investigation, writing – review and editing. **Runar Kjær:** investigation, writing – review and editing. **Svein Wara:** investigation, writing – review and editing. **Mikhail Mastepanov:** conceptualization, data curation, funding acquisition, investigation, methodology, supervision, validation, writing – review and editing.

## Conflicts of Interest

The authors declare no conflicts of interest.

## Supporting information


**Figure S1:** The experiment design at the cultivated peatland site in Pasvik.
**Figure S2:** The automatic chamber design.
**Figure S3:** Seasonal variations of the observed CO_2_ fluxes across 5 plots (vertically) during 2022–2023 (horizontally). Black vertical lines indicate the fertilization dates and yellow vertical lines indicate the harvest dates.
**Figure S4:** Seasonal variations of the observed CH_4_ fluxes across 5 plots (vertically) during 2022–2023 (horizontally). Black vertical lines indicate the fertilization dates and yellow vertical lines indicate the harvest dates.
**Figure S5:** Seasonal variations of the observed N_2_O fluxes across 5 plots (vertically) during 2022–2023 (horizontally). Black vertical lines indicate the fertilization dates and yellow vertical lines indicate the harvest dates.
**Figure S6:** Random Forest model predicted GHG fluxes as functions of the observed fluxes. The black solid lines indicate the 1:1 ratio.
**Figure S7:** Random Forest model predicted CO_2_ fluxes as functions of the observed fluxes during nighttime (global radiation < 10 W m^−2^) for different months. The black solid lines indicate the 1:1 ratio and the blue lines are the linear regression lines. Grey vertical bars indicate the SD of model predictions from 600 bootstrap samples.
**Table S1:** Variances associated with replicates (Rep) versus bootstrap (Bstrap) sampling for the estimated GHG budgets (t gas ha^−1^).

## Data Availability

The data and code are publicly available on Zenodo (DOI: https://doi.org/10.5281/zenodo.17423612).
