# Supplementary material for: Substantial Mitigation Potential for Greenhouse Gases Under High Water Levels in a Cultivated Peatland in the Arctic
Source: Glob Chang Biol. 2025 Nov 10;31(11):e70599. doi: 10.1111/gcb.70599 (PMC12598909; doi:10.1111/gcb.70599)
Supplement: Supplementary file 1 — Figure S1: The experiment design at the cultivated peatland site in Pasvik. Figure S2: The automatic chamber design. Figure S3: Seasonal variations of the observed CO2 fluxes across 5 plots (vertically) during 2022–2023 (horizontally). Black vertical lines indicate the fertilization dates and yellow vertical lines indicate the harvest dates. Figure S4: Seasonal variations of the observed CH4 fluxes across 5 plots (vertically) during 2022–2023 (horizontally). Black vertical lines indicate the fertilization dates and yellow vertical lines indicate the harvest dates. Figure S5: Seasonal variations of the observed N2O fluxes across 5 plots (vertically) during 2022–2023 (horizontally). Black vertical lines indicate the fertilization dates and yellow vertical lines indicate the harvest dates. Figure S6: Random Forest model predicted GHG fluxes as functions of the observed fluxes. The black solid lines indicate the 1:1 ratio. Figure S7: Random Forest model predicted CO2 fluxes as functions of the observed fluxes during nighttime (global radiation < 10 W m−2) for different months. The black solid lines indicate the 1:1 ratio and the blue lines are the linear regression lines. Grey vertical bars indicate the SD of model predictions from 600 bootstrap samples. Table S1: Variances associated with replicates (Rep) versus bootstrap (Bstrap) sampling for the estimated GHG budgets (t gas ha−1). [file GCB-31-e70599-s001.docx]

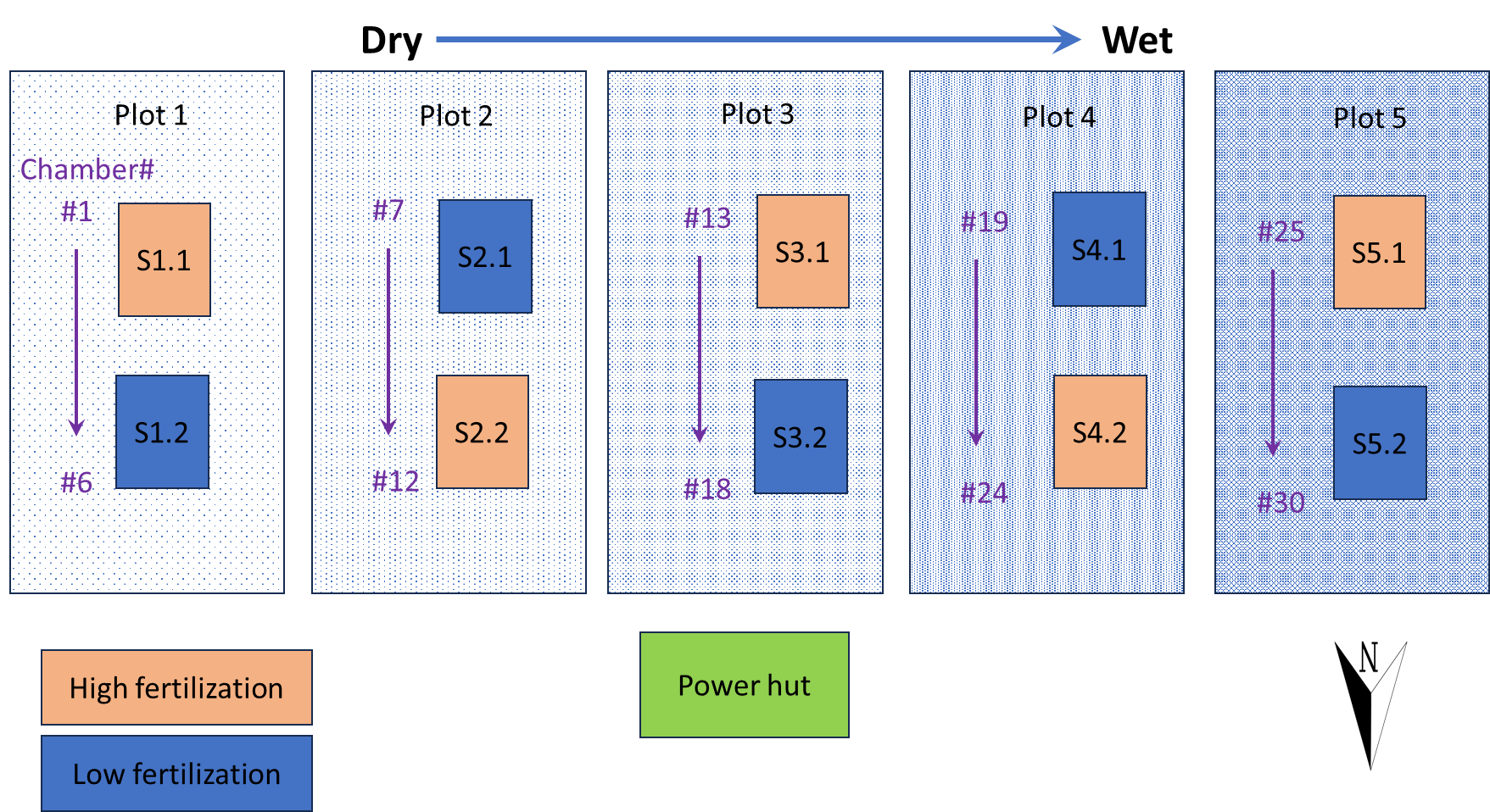


**Fig. S1** The experiment design at the cultivated peatland site in Pasvik.


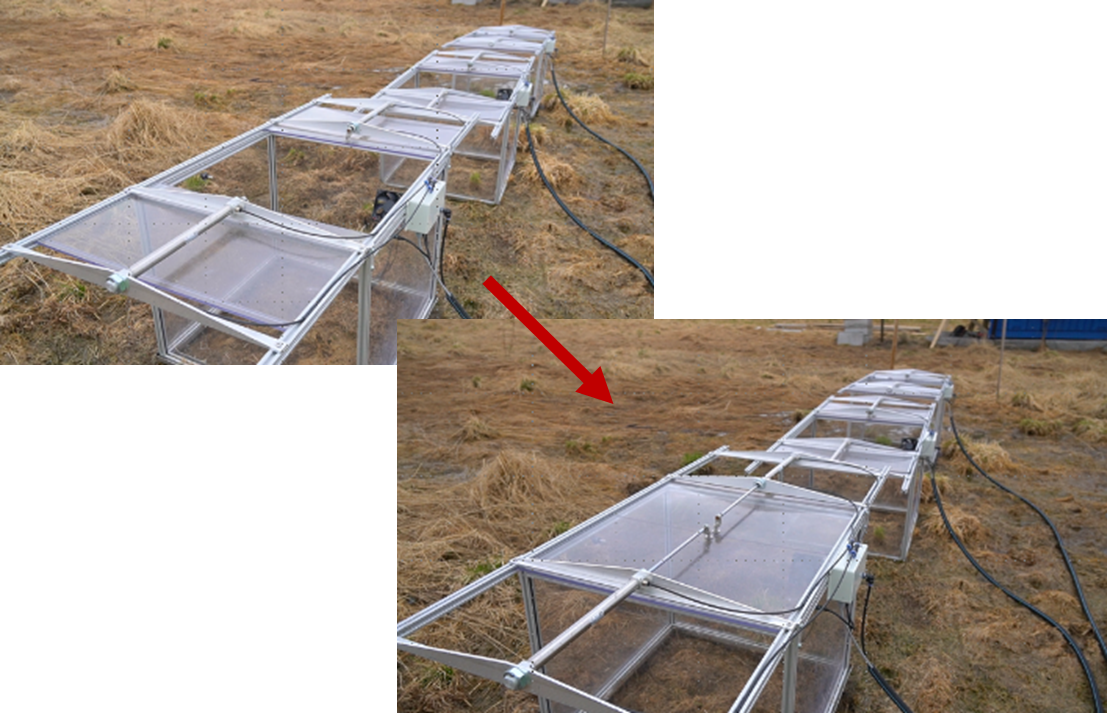


**Fig. S2** The automatic chamber design.


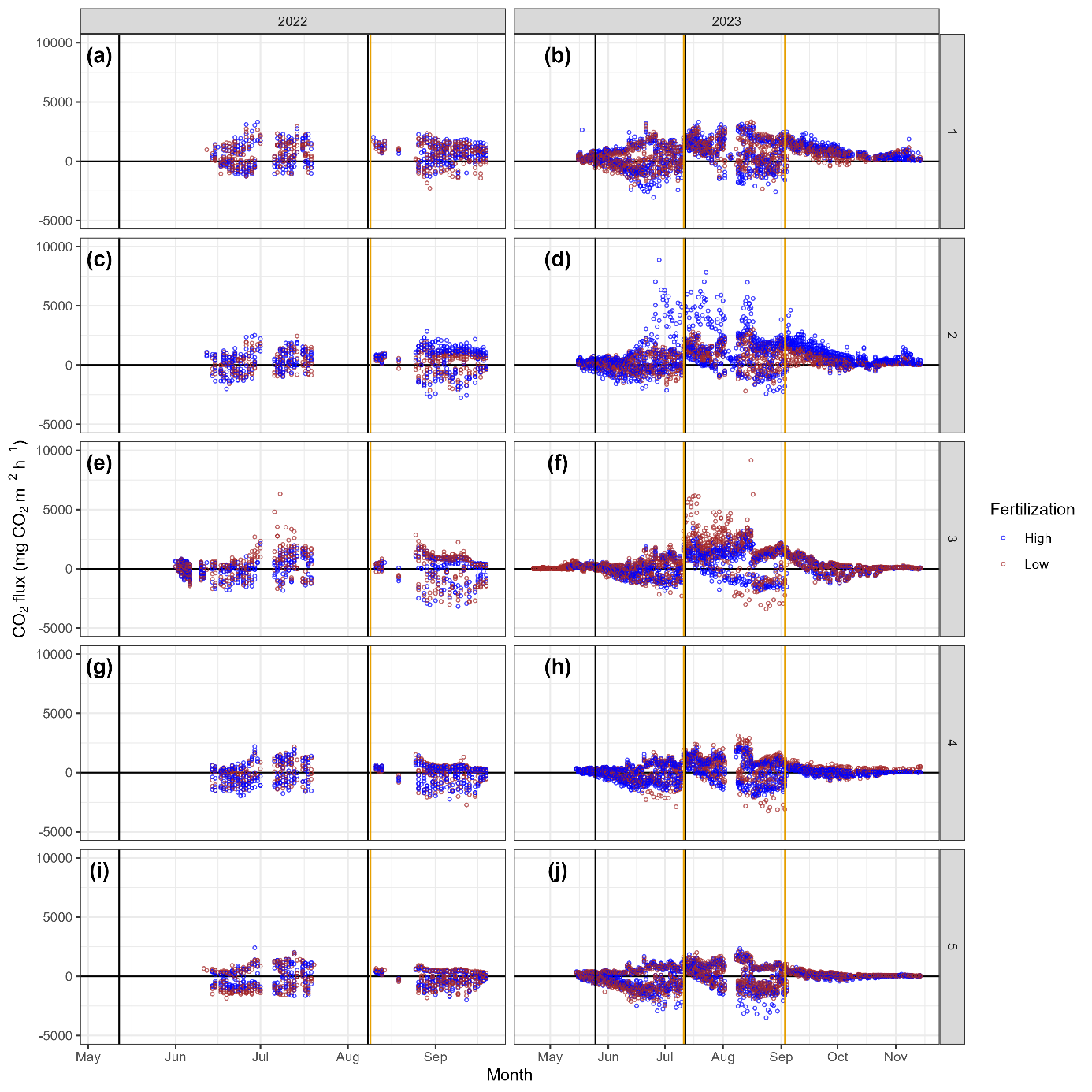


**Fig. S3** Seasonal variations of the observed CO_2_ fluxes across 5 plots (vertically) during 2022-2023 (horizontally). Black vertical lines indicate the fertilization dates and yellow vertical lines indicate the harvest dates.


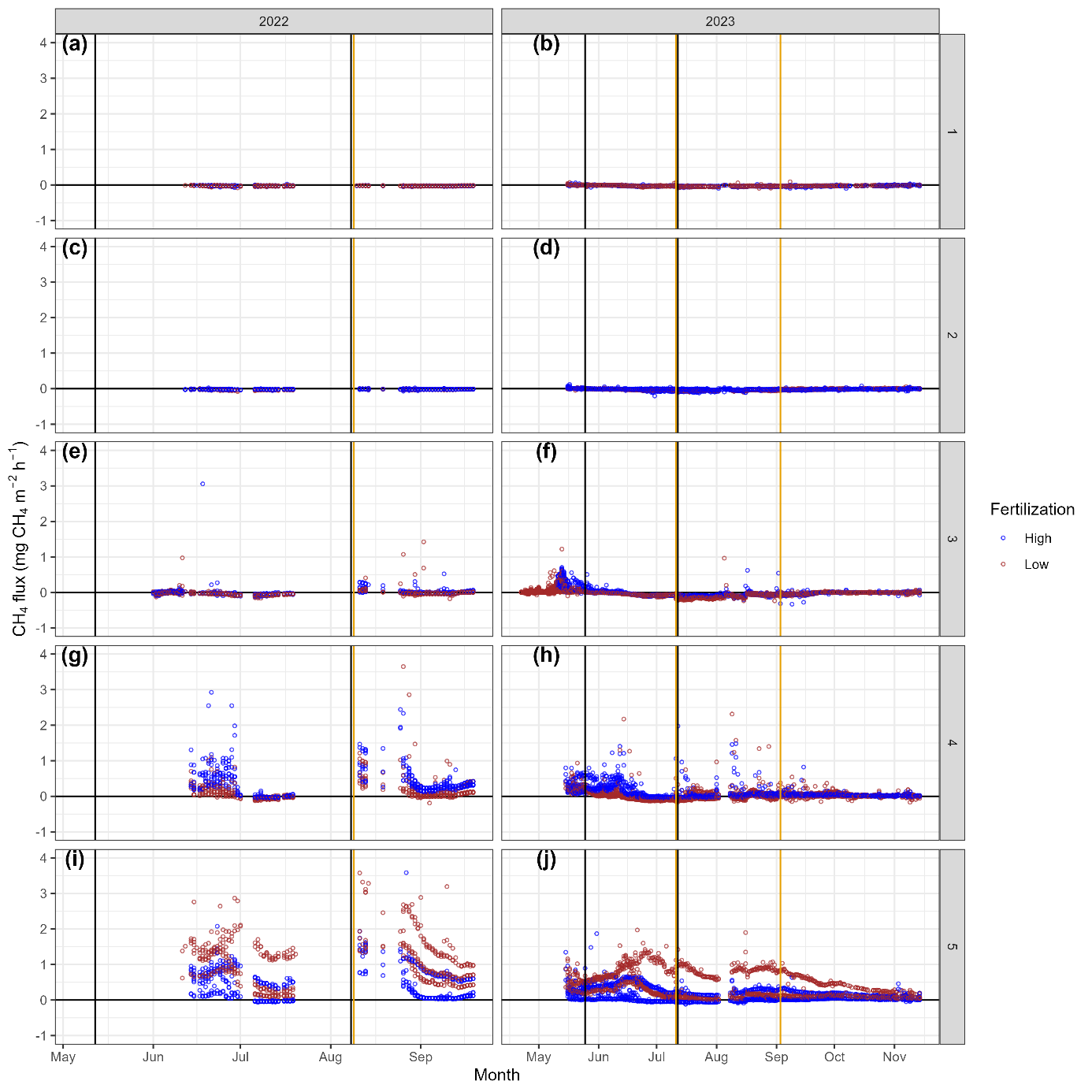


**Fig. S4** Seasonal variations of the observed CH_4_ fluxes across 5 plots (vertically) during 2022-2023 (horizontally). Black vertical lines indicate the fertilization dates and yellow vertical lines indicate the harvest dates.


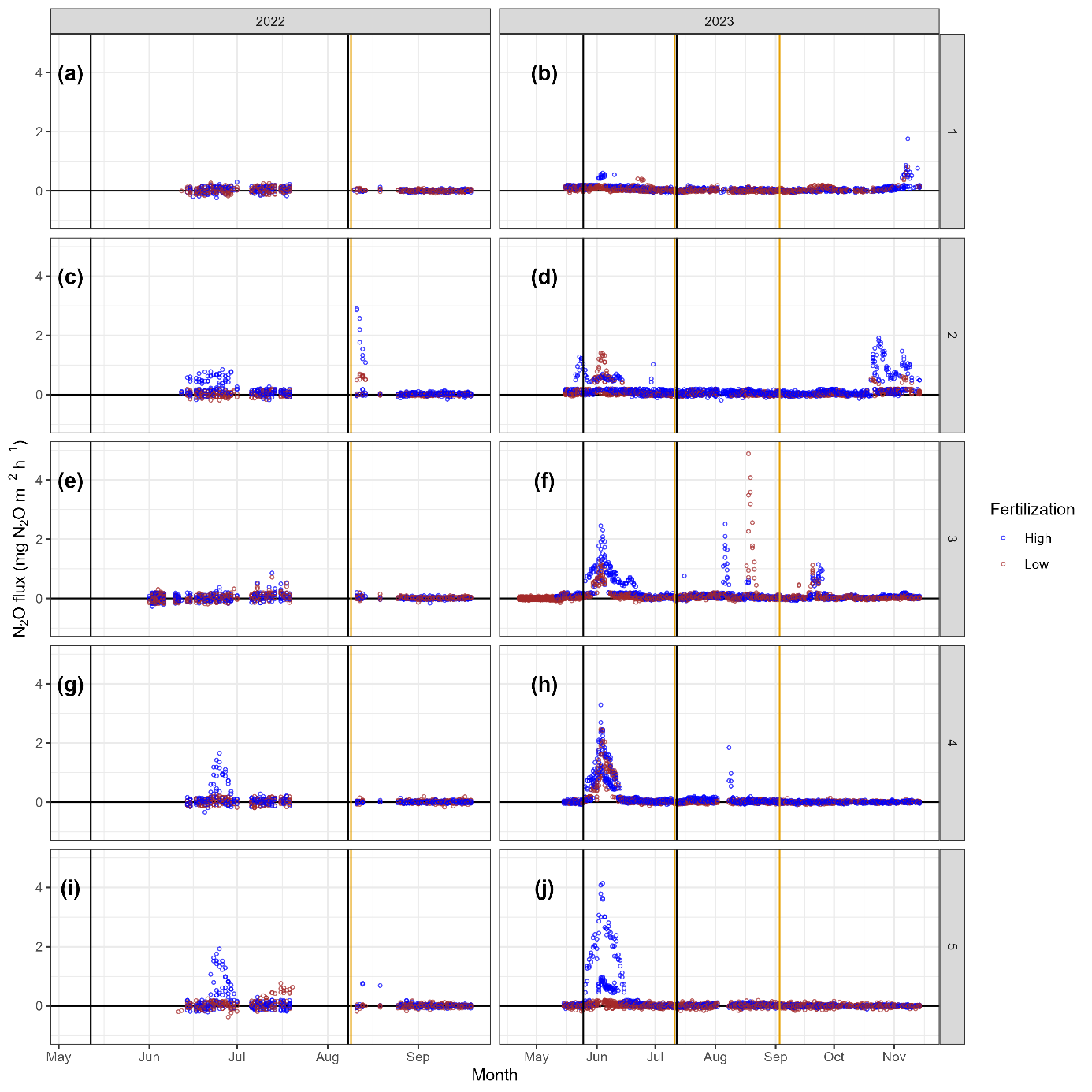


**Fig. S5** Seasonal variations of the observed N_2_O fluxes across 5 plots (vertically) during 2022-2023 (horizontally). Black vertical lines indicate the fertilization dates and yellow vertical lines indicate the harvest dates.


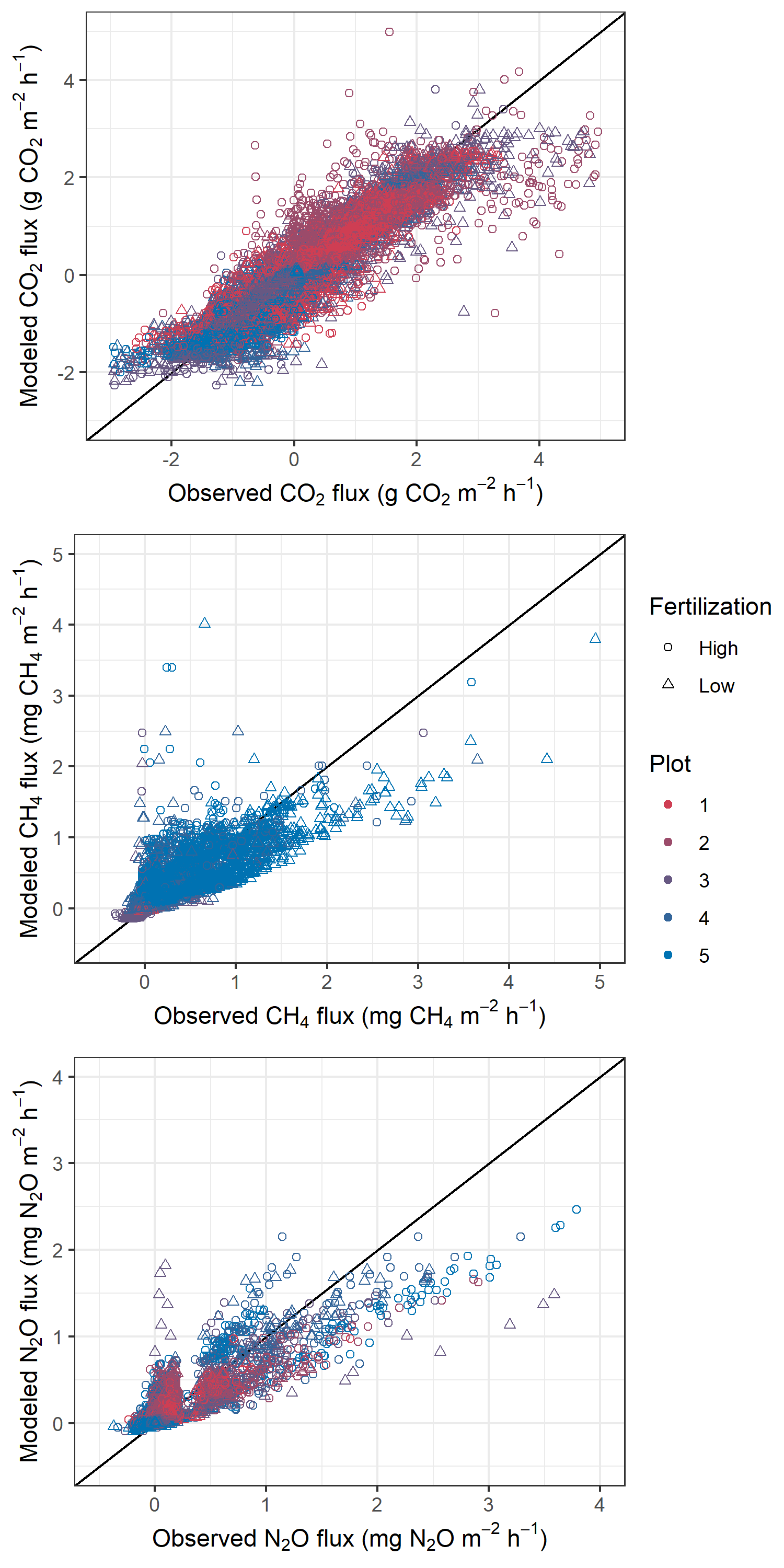


**Fig. S6** Random Forest model predicted GHG fluxes as functions of the observed fluxes. The black solid lines indicate the 1:1 ratio.


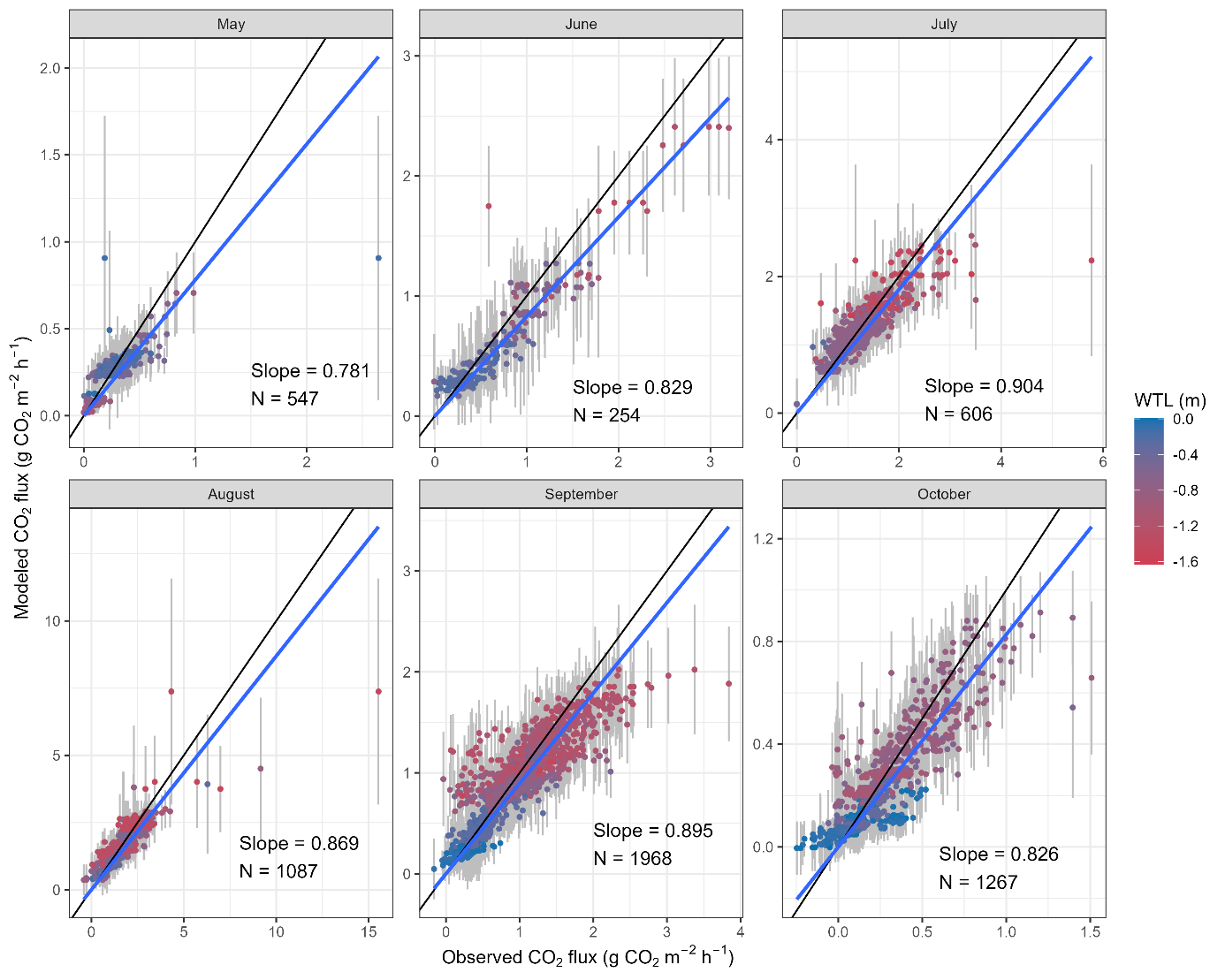


**Fig. S7** Random Forest model predicted CO_2_ fluxes as functions of the observed fluxes during nighttime (global radiation < 10 W m^-2^) for different months. The black solid lines indicate the 1:1 ratio and the blue lines are the linear regression lines. Grey vertical bars indicate the SD of model predictions from 600 bootstrap samples.

| **Table S1** Variances associated with replicates (Rep) versus bootstrap (Bstrap) sampling for the estimated GHG budgets (t gas ha^-1^) | | | | | | | | | | |
| --- | --- | --- | --- | --- | --- | --- | --- | --- | --- | --- |
|  |  |  | CO_2_ | |  | CH_4_ | |  | N_2_O | |
| Plot # | Fertilization | Year | Rep | Bstrap |  | Rep | Bstrap |  | Rep | Bstrap |
| 1 | High | 2022 | 2.84×10^-2^ | 7.27 |  | <1.00×10^-10^ | 1.30×10^-6^ |  | 2.00×10^-10^ | 2.5×10^-6^ |
|  |  | 2023 | 0.16 | 1.63 |  | 3.00×10^-10^ | 0.36×10^-6^ |  | 2.50×10^-9^ | 0.16×10^-6^ |
|  | Low | 2022 | 0.043 | 6.49 |  | <1.00×10^-10^ | 1.35×10^-6^ |  | 2.00×10^-10^ | 1.84×10^-6^ |
|  |  | 2023 | 0.44 | 1.90 |  | 1.00×10^-10^ | 0.34×10^-6^ |  | 6.00×10^-10^ | 0.07×10^-6^ |
| 2 | High | 2022 | 0.086 | 8.40 |  | <1.00×10^-10^ | 1.66×10^-6^ |  | 2.85×10^-8^ | 5.54×10^-6^ |
|  |  | 2023 | 11.6 | 4.64 |  | 1.40×10^-9^ | 0.22×10^-6^ |  | 5.14×10^-8^ | 0.32×10^-6^ |
|  | Low | 2022 | 0.021 | 6.22 |  | 1.00×10^-10^ | 1.73×10^-6^ |  | 1.30×10^-9^ | 2.59×10^-6^ |
|  |  | 2023 | 0.022 | 3.71 |  | 2.00×10^-10^ | 0.19×10^-6^ |  | 1.89×10^-8^ | 0.22×10^-6^ |
| 3 | High | 2022 | 0.020 | 5.32 |  | 1.50×10^-9^ | 2.26×10^-6^ |  | 1.00×10^-9^ | 3.63×10^-6^ |
|  |  | 2023 | 0.017 | 1.17 |  | 2.80×10^-8^ | 0.09×10^-6^ |  | 3.11×10^-8^ | 0.35×10^-6^ |
|  | Low | 2022 | 0.12 | 6.15 |  | 2.00×10^-9^ | 2.22×10^-6^ |  | 5.00×10^-10^ | 2.23×10^-6^ |
|  |  | 2023 | 0.17 | 1.51 |  | 2.33×10^-6^ | 0.05×10^-6^ |  | 3.32×10^-8^ | 0.25×10^-6^ |
| 4 | High | 2022 | 0.013 | 4.45 |  | 6.10×10^-8^ | 4.33×10^-6^ |  | 8.50×10^-9^ | 3.95×10^-6^ |
|  |  | 2023 | 0.003 | 1.50 |  | 1.80×10^-9^ | 1.88×10^-6^ |  | 5.00×10^-8^ | 0.23×10^-6^ |
|  | Low | 2022 | 0.002 | 5.00 |  | 1.82×10^-8^ | 7.37×10^-6^ |  | 2.00×10^-10^ | 2.67×10^-6^ |
|  |  | 2023 | 0.18 | 1.46 |  | 3.24×10^-8^ | 1.31×10^-6^ |  | 7.40×10^-9^ | 0.14×10^-6^ |
| 5 | High | 2022 | 2.73×10^-3^ | 4.46 |  | 2.49×10^-7^ | 9.66×10^-6^ |  | 8.60×10^-9^ | 5.28×10^-6^ |
|  |  | 2023 | 0.057 | 2.51 |  | 3.77×10^-7^ | 4.82×10^-6^ |  | 1.23×10^-7^ | 0.66×10^-6^ |
|  | Low | 2022 | 6.14×10^-3^ | 4.53 |  | 4.20×10^-7^ | 1.28×10^-5^ |  | 1.70×10^-9^ | 2.6×10^-6^ |
|  |  | 2023 | 0.19 | 2.40 |  | 1.19×10^-6^ | 4.92×10^-6^ |  | 1.00×10^-9^ | 0.6×10^-6^ |
|  |  |  |  |  |  |  |  |  |  |  |
|  |  | Mean | 0.66 | 4.04 |  | 2.36×10^-7^ | 2.94×10^-6^ |  | 1.85×10^-8^ | 1.79×10^-6^ |
|  |  |  |  |  |  |  |  |  |  |  |
